# Trophic Ecology of Atlantic Bluefin Tuna (*Thunnusthynnus*) Larvae from the Gulf of Mexico and NW Mediterranean Spawning Grounds: A Comparative Stable Isotope Study

**DOI:** 10.1371/journal.pone.0133406

**Published:** 2015-07-30

**Authors:** Raúl Laiz-Carrión, Trika Gerard, Amaya Uriarte, Estrella Malca, José María Quintanilla, Barbara A. Muhling, Francisco Alemany, Sarah L. Privoznik, Akihiro Shiroza, John T. Lamkin, Alberto García

**Affiliations:** 1 Instituto Español de Oceanografía—Centro Oceanográfico de Málaga (COM-IEO), Fuengirola, Spain; 2 Southeast Fisheries Science Center, National Marine Fisheries Service, National Oceanic and Atmospheric Administration (NOAA), Miami, Florida, United States of America; 3 South Florida Campus- University of Phoenix, Miramar, Florida, United States of America; 4 Cooperative Institute for Marine and Atmospheric Studies (CIMAS), University of Miami, Miami, Florida, United States of America; 5 Princeton University Program in Atmospheric and Oceanic Science, Forrestal Campus/Sayre Hall, Princeton, New Jersey, United States of America; 6 NOAA Geophysical Fluid Dynamics Laboratory, 201 Forrestal Road, Princeton, New Jersey, United States of America; 7 Instituto Español de Oceanografía Centro Oceanográfico de Baleares (COB-IEO), Palma de Mallorca, Balearic Islands, Spain; Aristotle University of Thessaloniki, GREECE

## Abstract

The present study uses stable isotopes of nitrogen and carbon (δ^15^Nandδ^13^C) as trophic indicators for Atlantic bluefin tuna larvae (BFT) (6–10 mm standard length) in the highly contrasting environmental conditions of the Gulf of Mexico (GOM) and the Balearic Sea (MED). These regions are differentiated by their temperature regime and relative productivity, with the GOM being significantly warmer and more productive. MED BFT larvae showed the highest δ^15^N signatures, implying an elevated trophic position above the underlying microzooplankton baseline. Ontogenetic dietary shifts were observed in the BFT larvae from the GOM and MED which indicates early life trophodynamics differences between these spawning habitats. Significant trophic differences between the GOM and MED larvae were observed in relation to δ^15^N signatures in favour of the MED larvae, which may have important implications in their growth during their early life stages.These low δ^15^N levels in the zooplankton from the GOM may be an indication of a shifting isotopic baseline in pelagic food webs due to diatrophic inputs by cyanobacteria. Lack of enrichment for δ^15^N in BFT larvae compared to zooplankton implies an alternative grazing pathway from the traditional food chain of phytoplankton—zooplankton—larval fish. Results provide insight for a comparative characterization of the trophic pathways variability of the two main spawning grounds for BFT larvae.

## Introduction

Atlantic bluefin tuna (*Thunnus thynnus*) (BFT) has the largest geographical distribution of any large pelagic fish living in temperate Atlantic waters [1–2]. This species reproduces in two geographically separate spawning regions: the Gulf of Mexico (GOM) and in the Mediterranean Sea (MED). Tagging studies have shown that BFT have spawning site fidelity to either the MED or the GOM ([3–4], supporting a hypothesis of homing behavior to spawning grounds [5–6]. This spawning segregation led fisheries assessment managers to distinguish between a western and eastern BFT stock [3], [6]. While spawning in the GOM takes place from April to June, spawning in the MED occurs from June to August [6–9].

Spawning grounds in the GOM and the MED show particular bio-physical and climatic characteristics [9–13]. The main oceanographic driving force in the GOM is the Loop Current, which flows from the Yucatan Straits into the eastern GOM, originating a strong anticyclonic flow. Meanders and frontal eddies associated with the Loop Current create areas of positive and negative vorticity that generate retention and enhanced production areas [14], influencing BFT spawning [15–16]. The western GOM is indirectly influenced by the Loop Current through mesoscale anticyclonic and cyclonic eddies, which detach and are driven westward from the Loop Current [17]. Cyclonic eddies may have enhanced primary and secondary production compared to surrounding oligotrophic waters [18–22]. The GOM can thus essentially be divided into two regions on the basis of oceanographic features: eastern (E-GOM) and western (W-GOM). Adult BFT in the GOM are found preferentially in the shelf break region of the western GOM, in areas with surface temperatures ranging from 24–27°C and relatively low chlorophyll (<0.16 mg m^-3^)[9]. Similarly, BFT larval abundance is higher in the western part of the GOM [23], [10].

The Balearic Sea is amongst the most important BFT spawning areas in the MED. The spawning grounds are strongly linked to the hydrographic features that characterize the Balearic Sea [11], [12], [24]. The area is under the influence of incoming Atlantic surface waters encountering resident surface water masses, which results in a complex hydrography characterized by frontal structures and associated mesoscales features, such as anticyclonic and cyclonic gyres [25–27]. BFT spawning in the area appears to be associated with a surface temperature range of ~ 21.5–26.5°C [12], [28].

When comparing the GOM and Balearic Sea spawning habitats, some common features stand out. Both are characterized by warm temperature regimes (21.5–28°C) in open sea regions, where chlorophyll production is low and where a series of mesoscale hydrographic features, such as frontal systems and eddy formation, occur. These mesoscale structures may provide conditions matching the “ocean triad” hypothesis (enrichment, concentration, retention) [14], [29] and facilitate the concentration of both, food particles and BFT larvae [11]. Thus, higher BFT larval abundances appear to be linked to anticyclonic gyres or eddies in the Balearic Sea, south of the island of Menorca [28] and to the boundaries of anticyclonic eddies in the GOM [30].

Although several previous studies have described the distributions and environmental associations of BFT larvae, very little is known regarding their ecology, and the primary processes influencing larval survival in oligotrophic spawning grounds. Previous trophic studies on larvae of apex predators, including BFT and other scombrids, have mainly relied on stomach content analysis, which only records the recently ingested prey [31]. The only study on stomach content analysis of BFT larvae from the MED showed that the main prey items were cladocerans and nauplii [32]. Moreover, unlike other tuna species from this area, such as *T*. *alalunga* and *Auxis thazard*, BFT larvae did not show a piscivorous diet. Other tuna larvae, such as *Auxis* spp., showed a preference for appendicularians [33–34]. These organisms, which constitute an essential part of the diet for tuna larvae in the Pacific Ocean, form part of the trophic link to the microbial loop [35].

This study aimed at gaining the first known insight into the trophodynamics of early life stages of BFT through a comparative spawning ecosystem approach using stable isotope analysis (SIA). The comparative trophic ecology of GOM and MED BFT larvae was assessed using SIA of larvae in relation to baseline feeding levels defined by the micro and the mesozooplankton size fractions. Currently, few studies have attempted to use these natural dietary tracers to examine the early life feeding ecology of pelagic fishes in marine ecosystems [36–39].

Trophic flow is analyzed using the heavy isotope of nitrogen, ^15^N. This tracer is enriched through successive trophic levels, thereby providing information on the trophic position of organisms ([40–42]. In addition, SIA can trace sources of nitrogen and N^2^ inputs, because atmospheric nitrogen is relatively depleted in heavy (^15^N) isotopes compared with marine nitrate [43]. Assimilation of this light N_2_ by diazotrophs produces organic matter with a characteristic isotopic signature that can be traced throughout the food web. Nitrogen isotopes in seston reflect the uptake of atmospheric N_2_ by cyanobacteria (e.g. [44]), while those in zooplankton show the assimilation of organic matter initially produced by diazotrophs [45].

On the other hand, the heavy isotope of carbon (^13^C) can be used for determining the energy sources of dietary carbon, since it varies significantly among primary producers which have different photosynthetic pathways. In contrast to ^15^N, it does not vary substantially with trophic transfers [41], [42], [46]. The SIA approach using ^15^N and ^13^C thus provides a more integrated view of trophic dynamics than stomach content analysis [47], as it analyzes species trophic interactions with assimilated food rather than ingested food [48–49].

This SIA study examines the early life trophodynamics of BFT by comparingδ^13^C and δ^15^N tracers as indicators of the trophic relationships between BFT larvae originating from different spawning grounds (GOM and MED). The possible dietary shifts during larval development and the differences observed in the nitrogen cycle of each BFT spawning region are discussed.

## Material and Methods

### Field collection of BFT larvae and planktonic sampling

**Fig 1:**
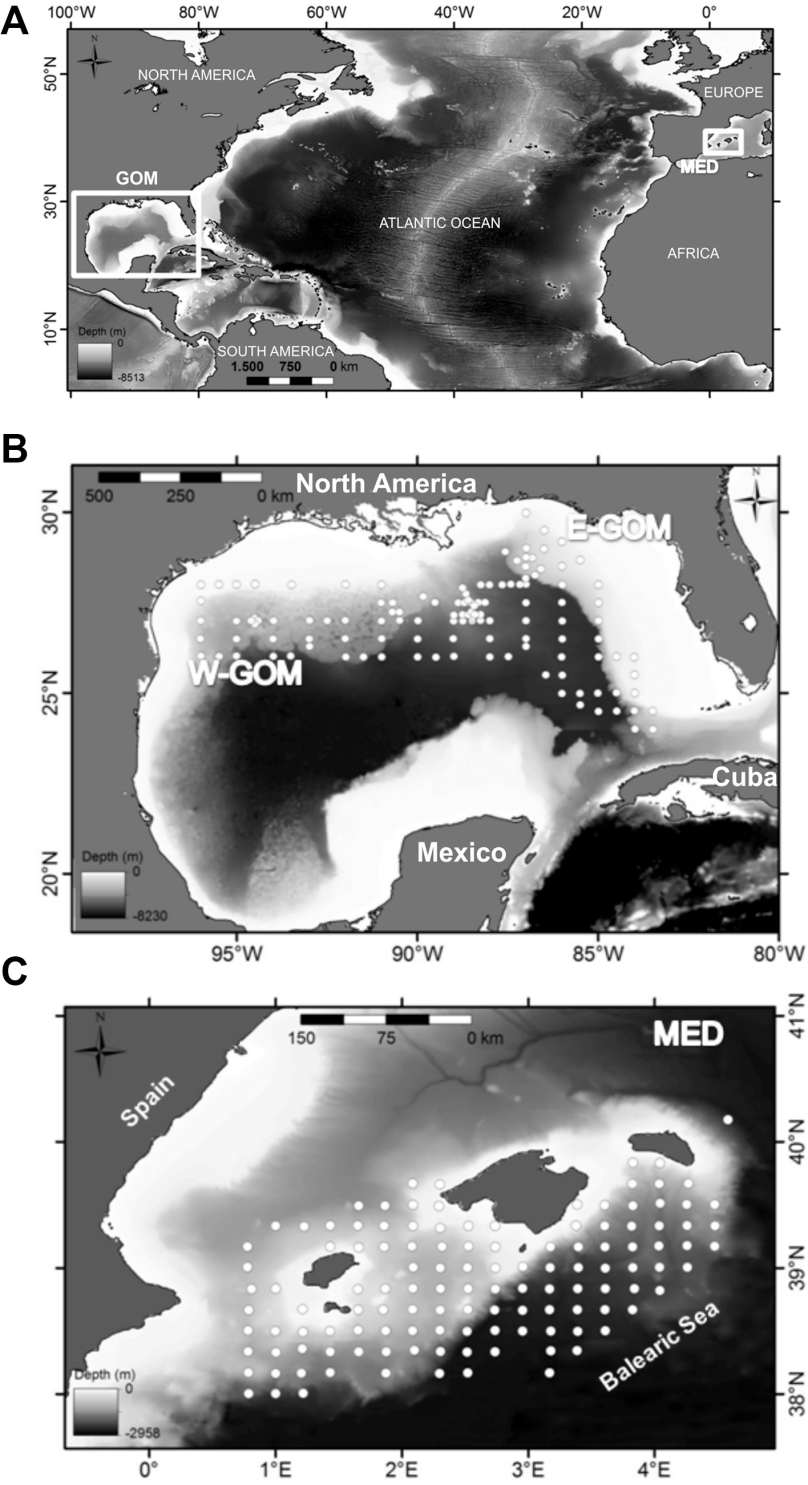
Location of the study areas. (A) Gulf of Mexico (GOM) and Balearic Sea (MED). (B) Eastern (E-GOM) and Western (W-GOM) and (C) MED bluefin tuna study area showing the oceanographic-planktonic stations sampling distribution. Bathymetric image generated from ETOPO1 database [51].

BFT larvae were collected in the two main reproductive areas for this species: the GOM and MED (Fig 1A). A total of 208 stations were sampled onboard NOAA’s R/V *Gordon Gunter*, of which 127 were completed in the GOM, during spring 2012, from April 24 to May 28, as part of an annual larval survey carried out by the National Marine Fisheries Service Southeast Area Monitoring and Assessment Program (SEAMAP) (Fig 1B). The MED BFT larvae were sampled from 124 systematic stations during summer 2013 (June19 to July 13) in the Balearic Sea, Western Mediterranean; (Fig 1C) onboard the R/V *SOCIB*. (See S1 Table for station’s geographical coordinates) Fish larvae were sampled using similar methodologies in both study areas. That is standard double oblique subsurface hauls using a 505μm mesh net attached to a standard 1 x 2 meter neuston frame in the GOM and a squared-mouth Bongo frame of 0.9 meter in the MED, fitted also with 505μm mesh size net. The nets were towed in an undulating manner through the upper 10–20 m of the water column for ten minutes per tow [39], [50]. General Oceanics 2030 flowmeters were placed at the center of the net’s mouth to calculate the water volume filtered. The research presented in this manuscript involved no endangered or protected species. No special permission was required for field sampling in the locations of the off shore study areas. Fish larvae included in the plankton samples are dead upon retrieval of the net No specific approval for this vertebrate scientific activity is required since ichthyoplankton sampling involved collecting deceased fish larvae.

**Fig 2:**
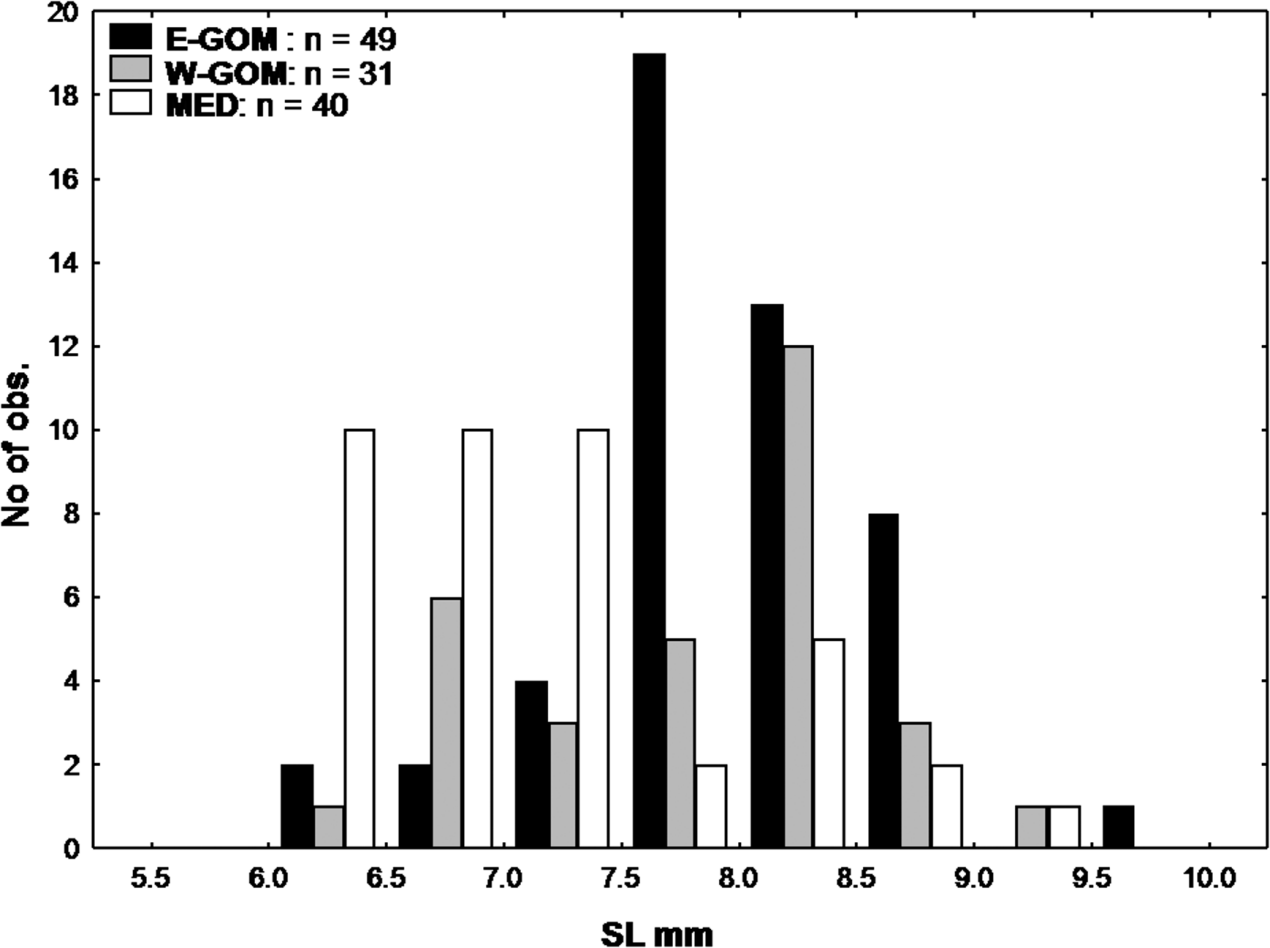
Bluefin tuna larval size frequency distributions. Eastern Gulf of Mexico (E-GOM), Western Gulf of Mexico (W-GOM) and Balearic Sea (MED) bluefin tuna larval size frequency distributions analyzed for trophic ecology studies.

Larvae were sorted from the plankton samples immediately after retrieval of the net and preserved frozen at -200°C onboard. A total of 119 BFT larvae were sorted onboard from the E-GOM, 109 from the W-GOM and 853 from the MED. In order to avoid maternally transmitted isotopes effects on preflexion BFT larvae [52], preflexion stages were excluded from the comparative trophic ecology analysis and only a similar size range of 6–10mm postflexion BFT larvae have been used in this study (Fig 2). From all sorted larvae, 49 from the E-GOM, 31 from W-GOM and 30 from the MED were respectively selected for stable isotope analysis as described in [39].

A 20 cm diameter Bongo net was geared above the neuston net to sample different zooplankton fractions by employing 55 and 200 μm mesh nets, each one equipped with General Oceanics flowmeters. Mesozooplankton (>200 μm) samples were divided into two aliquots using a Folsom plankton sample splitter. One sub-sample was preserved in 90% ethanol, and the other frozen for SIA. Samples from the 55 μm mesh nets were sieved on board to separate the microzooplankton (55 to 200 μm) fraction. These samples were also stored frozen at -20°C. Both microzooplankton and mesozooplankton samples were weighed to the nearest 1 μg on an electronic microbalance after freeze drying for 48 h at −20°C for biomass estimations. Dry weight biomass values were standardised to mg m^-3^.

Hydrographic data were collected at each sampling station using a Seabird 19+ CTD profiler cast to a minimum depth of 200 m for the GOM, or within 10m of the seabed at shallower stations,and 350 m or at least 5 m above the seabed for the MED.

### Isotopic analyses and trophic level calculation

In the laboratory, larvae were thawed to ambient temperature, measured to standard length (SL), dehydrated in a freeze dryer for 24 h and dry weighed (DW) on a precision microbalance (0.01mg) for subsequent SIA. Dry weighed larvae (0.08–1.9 mg) were packed in tin vials (0.03 ml) before analysis. Natural abundance of N (δ^15^N, %N) and C (δ^13^C, %C) were measured using an isotope-ratio spectrometer (Thermo-Finnigan Deltaplus; www.thermoscientific.com) coupled to an elemental analyser (FlashEA1112 Thermo- Finnigan) at the Instrumental Unit of Analysis of the University of A Coruña. Ratios of ^15^N:^14^N and ^12^C:^13^C were expressed in conventional delta notation (δ), relative to the international standard [atmospheric air (N_2_) and Pee-Dee Belemnite (PDB), respectively, using acetanilide as standard]. The accuracy of the measurements for δ^15^N and δ^13^C were 0.17 and 0.15‰, respectively.

Lipid correction was not possible due to the low amount of sample available, which hampered a previous chemical extraction. Nevertheless, a posterior correction of the δ^13^C values for lipid content was performed for the different plankton size fractions. The equations proposed by [53] for marine invertebrates were applied to select the best model predicting the lipid correction. The average of the three equations of the model were applied to estimate a value of 1.59‰ (SD = 0.41) and 0.82‰ (SD = 0.26) for micro- and meso-zooplankton, respectively. These values were used for the δ^13^C lipid correction in both zooplankton size fractions. The lipid correction for BFT larvae took into account the parameters of Atlantic BFT muscle reported by [53]. The mean value for lipid correction was 2.45 ‰ (SD = 0.44) for the GOM BFT larvae and 1.38 (SD = 0.38) for MED BFT larvae.

Trophic position (TP) for each BFT larvae was calculated following Eq (1):

$$TP=TP_{basal}+\frac{\delta^{15}N_{larva}-\delta^{15}N_{micro}}{\Delta^{15}N}$$ (1)

δ^15^N_larva_ were the isotopic signatures for individual BFT larvae and δ^15^N_micro_ were the isotopic values of microzooplankton of the larvae related station. We applied a basal trophic position (TP_basal_) of 2, assuming microzooplankton as primary consumers [49], [54]. The values of the mean nitrogen isotopic discrimination factor for (Δ^15^N) proposed by [55] for BFT juveniles were used (1.64 ‰).

### Statistical analysis

As the common size ranges of larval cohorts, ranging from 6 to 10 mm, showed significant statistical differences for SL and DW relationships, the analysis of covariance (ANCOVA) tests with SL as covariate were used. ANCOVA tests were applied to verify differences among regions for somatic variables (SL, DW), larval stable isotopes (δ^15^N, δ^13^C), N and C content (%N, %C and C:N) and trophic position. Logarithmic and arcsine transformations of the data were carried out when necessary to fulfil the conditions of ANOVA. The differences between groups for δ^15^N were analyzed using a 2-way crossed ANOVA with plankton size fractions (micro- and mesozooplankton and larvae) and regions (E-GOM, W-GOM and MED) as main factors. Post-hoc comparisons were made using a Fisher’s test. All statistical tests were performed using Statistica7.1 (Statsoft).

## Results

### GOM and MED BFT spawning habitat conditions

The hydrographic conditions of each BFT spawning grounds analyzed in this study showed differences in temperatures and salinities (Table 1). The average surface temperatures in the E-GOM and W-GOM were significantly higher than in the MED (over 2–3°C). Inversely, sea surface salinity was higher in the MED. Within the GOM, the W-GOM was warmer than the E-GOM. Temperature at 100m was also much warmer in the GOM than in the MED, where at 100m depth the water temperature was around 13°C, which are those found throughout the water column during the non stratified winter period, since the thermocline during the stratified water column period, which lasts from the end of the spring to early autumn only reach around 50 m depth. The Balearic Sea scenario was characterized by the presence of a density front resulting from the encounter of water masses with distinct salinity, the saltier resident Atlantic water and the fresher Atlantic water, flowing directly from the Atlantic Ocean.

**Table 1:** Basic hydrographic data of the study area.

	5 m depth					
	Temperature °C			Salinity ‰		
	Mean±StdDv	Max.	Min.	Mean±StdDv	Max.	Min.
E-GOM	25.51 ± 0.55	26.13	24.63	36.07 ± 0.23	36.34	35.64
W-GOM	26.65 ± 0.59	27.25	25.95	36.34 ± 0.32	36.63	36.03
MED	22.98 ± 0.68	23.97	21.76	37.72 ± 0.16	38.12	37.51
	100 m depth					
	Temperature °C			Salinity ‰		
	Mean±StdDv	Max.	Min.	Mean±StdDv	Max.	Min.
E-GOM	19.99 ± 0.74	20.95	19.16	36.43 ± 0.15	36.58	36.18
W-GOM	20.90 ± 0.39	21.31	20.43	36.50 ± 0.03	36.52	36.45
MED	13.40 ± 0.15	13.64	13.14	38.24 ± 0.09	38.39	38.09

Temperature (°C) and salinity (‰)of the selected stations from the Eastern Gulf of Mexico (E-GOM) Western Gulf of Mexico (W-GOM) and Balearic Sea (MED) sampling regions.

**Fig 3:**
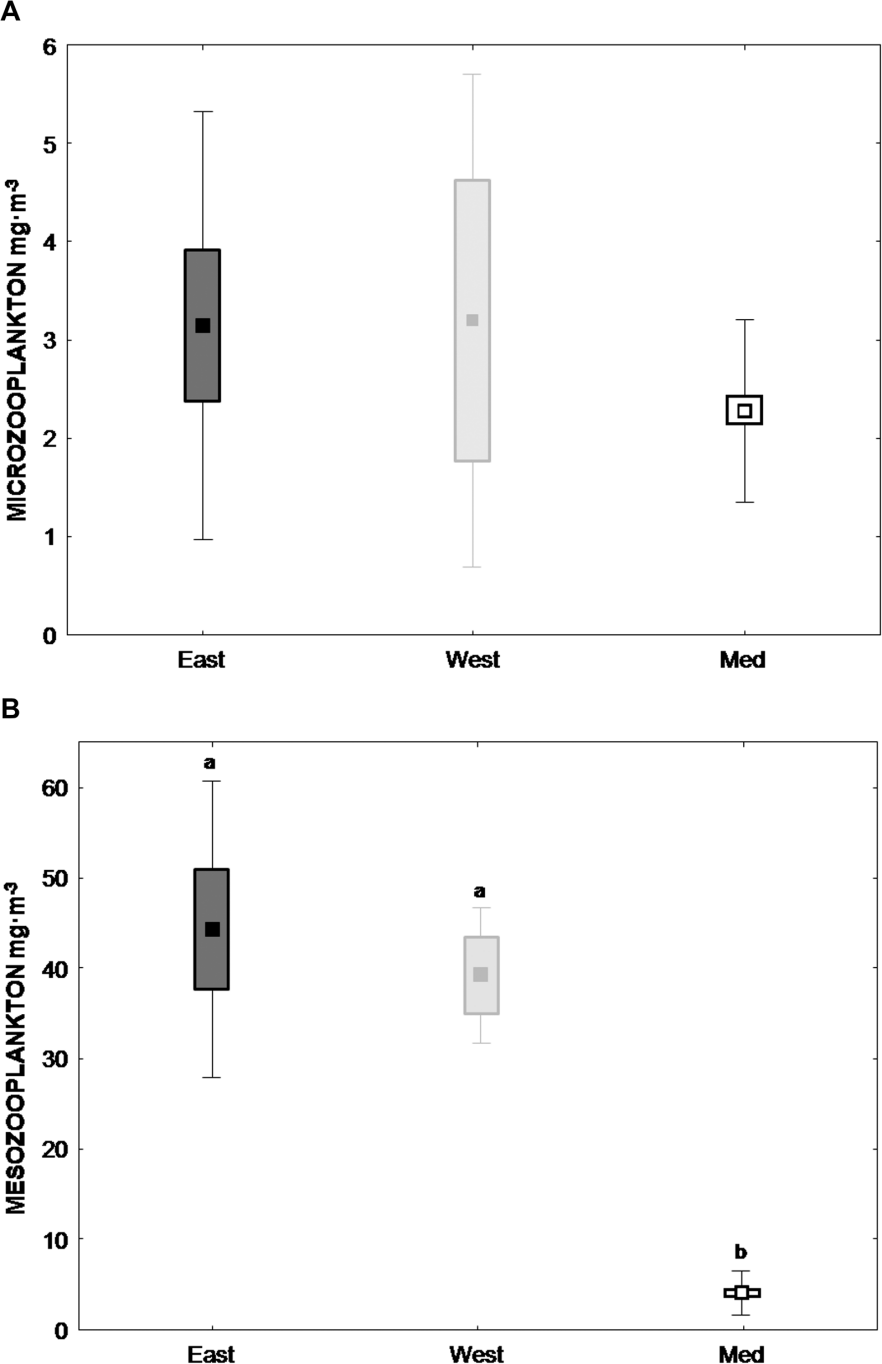
Biomass of zooplankton size fractions. One-way ANOVA analyses of both (A) microzooplankton [MICRO,55−200 μm]- and (B) mesozooplankton [MESO, >200 μm] size fraction biomass (mg·m^-3^) in Eastern Gulf of Mexico (E-GOM) (●), Western Gulf of Mexico (W-GOM) (●) and Balearic Sea (MED) (○) regions. Post-hoc comparisons were made using a Tukey’s test. Different letters indicate a significant difference between ecosystems. Box represents std error and whisker Std dev respectively.

Both, micro- and mesozooplankton biomass were higher in the GOM than in the MED (Fig 3). The greatest differences were found in the mesozooplankton fraction (ANOVA p< 0.001), where the mean mesozooplankton biomass in the GOM was up to 10 times greater than that recorded in the MED.

**Fig 4:**
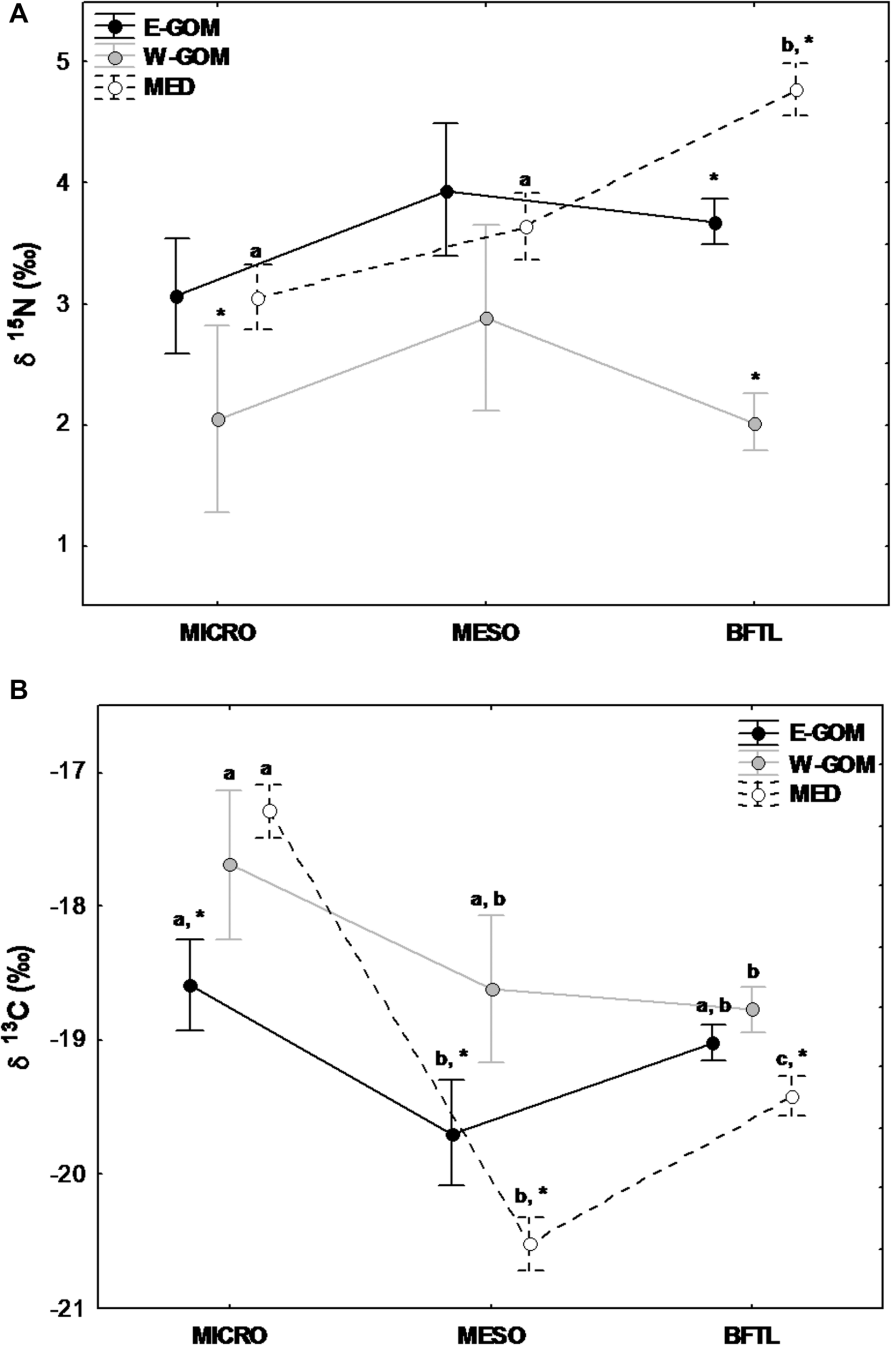
Mean nitrogen and carbon isotopic signature in MICRO, MESO and BFTL. δ^15^N (A) and δ^13^C (B) isotopic signature (Mean±SE) for different planktonic compartments analyzed (microzooplankton [MICRO,55−200 μm], mesozooplankton [MESO, >200 μm] and bluefin tuna larvae [BFTL]) in Eastern Gulf of Mexico (E-GOM) (●), Western Gulf of Mexico (W-GOM) (●) and Balearic Sea (MED) (○) regions. Regions (E-GOM, W-GOM and MED) and plankton size fraction (MICRO, MESO and BFTL) were the main factors for the 2-way ANOVA analysis. Post-hoc comparisons were made using a Tukey’s test. Different letters indicate significant differences (*p*< 0.05) among plankton size fractions within same region. * denotes a significant difference (*p*< 0.05) between regions for the same plankton size fraction.

The W-GOM microzooplankton fraction showed the lowest δ^15^N signatures among regions (ANOVA, p<0.001)(Fig 4A) whereas the lowest micro δ^13^C values were recorded in the E-GOM region. (ANOVA, p<0.001) (Fig 4B). In contrast, while δ^15^N signatures did not show significant differences in the mesozooplankton fraction among the spawning regions (ANOVA, p>0.05), δ^13^C showed lowest δ^13^C signatures in the MED mesozooplankton fraction, followed by the E- and W-GOM regions, respectively (ANOVA, p<0.001) (Fig 4A and 4B).

### Isotopic signatures from plankton to BFT larvae

The isotopic signatures of δ^15^N in the microzooplankton (55−200μm) and mesozooplankton fractions (>200μm) showed no statistical differences between the three regions considered; the E-GOM, the W-GOM and the MED (Fig 4A). However, the δ^15^N signatures in BFT larvae from the three areas showed important differences between them (two-way ANOVA, F4, 178 = 11.1; p <0.001). The MED BFT larvae showed the greatest trophic enrichment of δ^15^N in comparison to micro and mesozooplankton, while both the E- and W-GOM BFT larvae showed similar δ^15^N signatures to that of the zooplankton size fractions.

The δ^13^C signature of the microzooplanktonin the E-GOM was significantly lower than in the W-GOM and MED (Fig 4B), whereas for the mesozooplankton size fraction, the MED showed significantly lower δ^13^C signatures than the E- and W-GOM. Similarly, the MED BFT larvae had significantly lower δ^13^C ratios than the GOM BFT larvae.

### BFT larval morphometrics and C:N ratio

The BFT larvae used for this study represented a selected fraction of the larvae collected at positive BFT stations of both MED and GOM surveys. The larval samples originated from 24, 8 and 4 positive stations for the MED, E-GOM and W-GOM, respectively.

**Fig 5:**
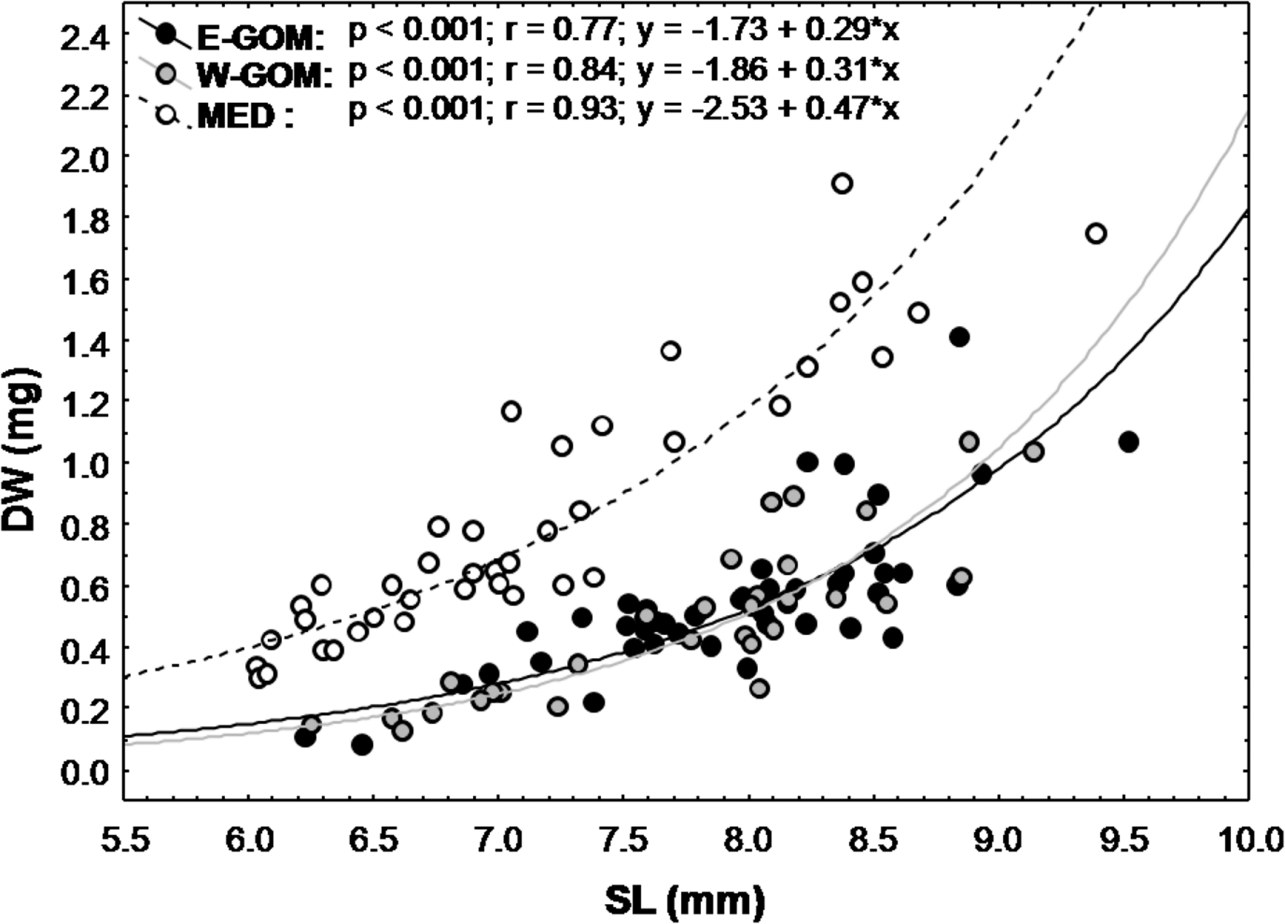
Dry weight-standard length BFTL relationships. Bluefin tuna larval dry weight (DW) *vs*. standard length (SL) relationships for Eastern Gulf of Mexico (E-GOM) (●), Western Gulf of Mexico (W-GOM) (●) and Balearic Sea (MED) (○) regions larval cohorts.

While no differences were observed between the E-GOM and the W-GOM BFT larval SL vs DW relationship, larvae from the MED and both GOM regions showed significant differences (Fig 5). The MED BFT larvae had a higher DW by SL (ANCOVA, F_2, 116_ = 125.5; *p*< 0.001) than either E-GOM or W-GOM larvae (ANCOVA, F_2, 116_ = 130.9; *p*< 0.001).

Higher C:N ratios were observed in GOM BFT larvae in comparison to the MED larvae (ANOVA, F_2, 115_ = 182.6; *p*< 0.001), while no statistical differences were observed between the E-GOM and W-GOM BFT larvae. Consequently, the δ^13^C lipid correction model showed significant differences between the GOM and MED BFT larvae (ANOVA F_2, 62_ = 58.8, *p*< 0.001)

### BFT larvae trophic relationship

**Fig 6:**
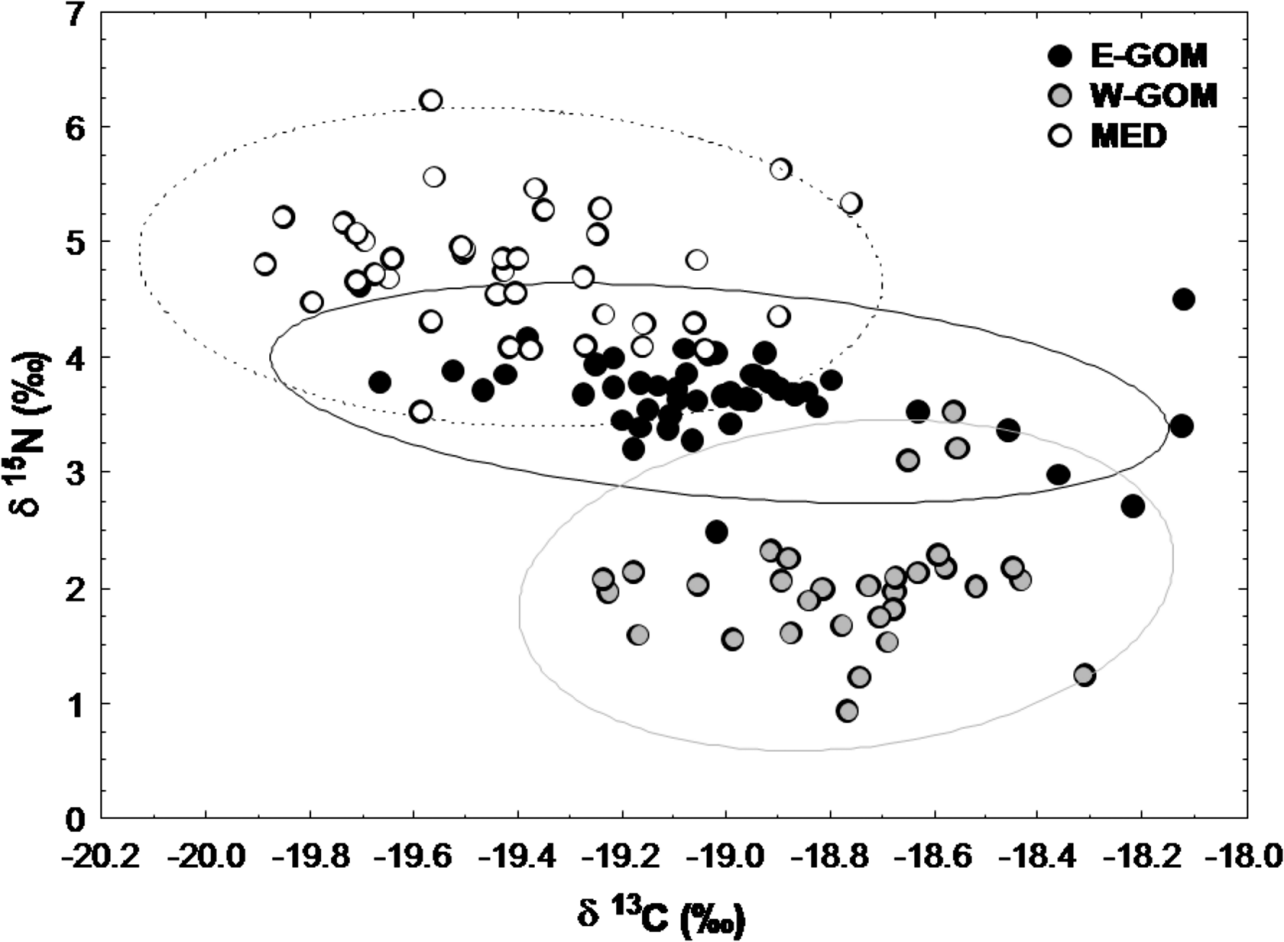
Nitrogen and carbon stable isotope scatterplot in bluefin tuna larvae. Relationship between δ^13^C and δ^15^N (‰) in bluefin tuna larvae in Eastern Gulf of Mexico (E-GOM) (●), Western Gulf of Mexico (W-GOM) (●) and Balearic Sea (MED) (○) regions. The ellipses represent the prediction interval with 0.95 coefficient.

The δ^15^N vs δ^13^C relationships of BFT larvae from the three studied spawning grounds showed clearly segregated δ^15^N signatures (Fig 6). On the other hand, the δ^13^C values were more similar among regions, with the W-GOM slightly more enriched than the E-GOM.

**Fig 7:**
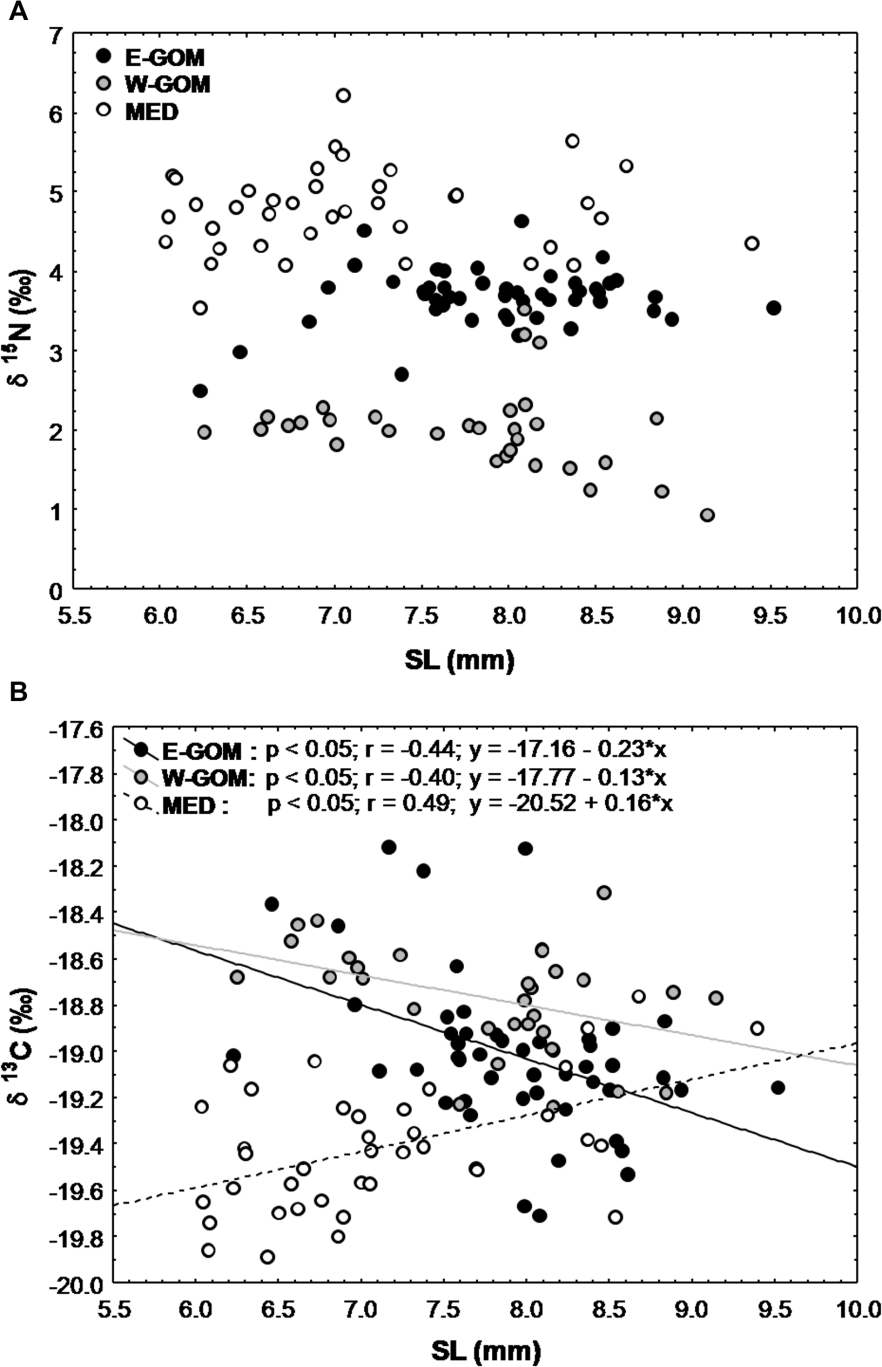
Nitrogen and carbon stable isotopes *vs* bluefin tuna larvae size relationship. Different δ^15^N (A) and δ^13^C (B) isotopic signature *vs* standard length (SL) relationships of bluefin tuna larvae in Eastern Gulf of Mexico (E-GOM) (●), Western Gulf of Mexico (W-GOM) (●) and Balearic Sea (MED) (○) regions.

No significant trends in δ^15^N larval signatures were observed in association withSL (Fig 7A). The highest δ^15^N values corresponded to the BFT larvae from the MED, followed by the E-GOM and W-GOM larvae. The signatures of δ^13^C of MED BFT were significantly lower than the GOM BFT larvae. However, the δ^13^C values of the MED BFT larvae showed a significant linear increase with SL (r = 0.49; p < 0.05; δ^13^C = -20.523 + 0.156·SL), while alternatively, these showed significant linear decrease in the E- and W-GOM BFT larvae (r = -0.44; p < 0.05; δ^13^C = -17.161–0.234·SL and r = -0.40; p < 0.05; δ^13^C = -17.766–0.129·SL, respectively) (Fig 7B).

The estimated Trophic Position (TP) of BFT larvae was significantly higher in the MED (3.00 ± 0.06) than in both the E-GOM (2.31 ± 0.06) and W-GOM (2.20 ± 0.07) (ANCOVA, F2, 112 = 39.8; p <0.001).

**Fig 8:**
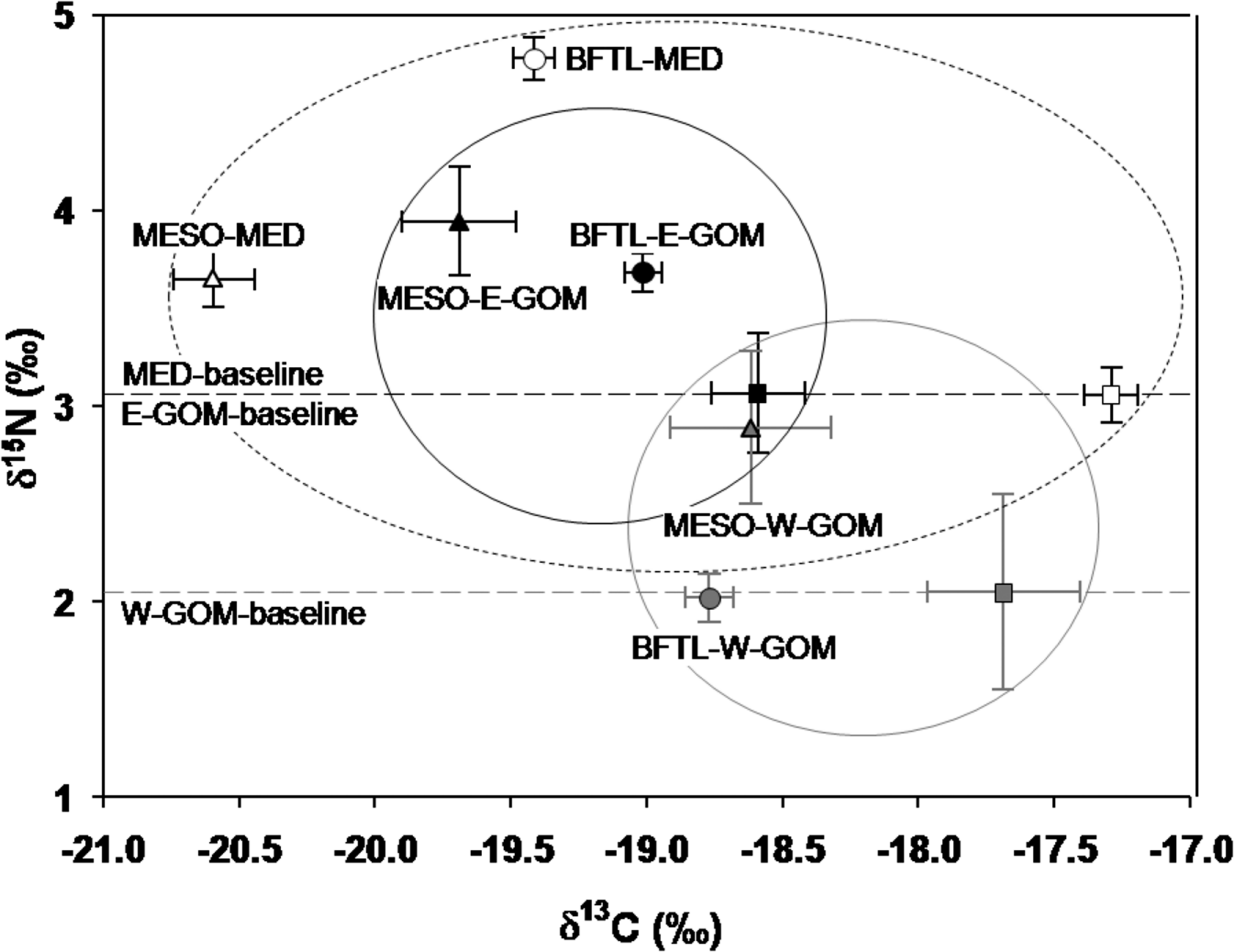
Biplot of mean nitrogen and carbon stable isotopes in bluefin tuna larvae. Mean (±SE) δ^13^C versus δ^15^N (‰) values mesozooplankton (MESO-triangles) and bluefin tuna larvae (BFTL-circles) in Eastern Gulf of Mexico (E-GOM) (●), Western Gulf of Mexico (W-GOM) (●) and Balearic Sea (MED) (○) regions. Microzooplankton as primary consumers has been used as baseline. The ellipses represent the isotopic variance for each region, encircling the isotopic signature for the different plankton size fractions (MICRO, MESO and BFTL).

The relationship between δ^15^N and δ^13^C in the mesozooplankton and BFT larvae together with the baselines levels are shown in Fig 8. In the MED region, BFT larvae showed the highest δ^15^N mean, located above the mesozooplankton fraction values and above the microzooplankton fraction signature in that order. In contrast, in the GOM region, the mean δ^15^N of BFT larvae from the E-GOM are in the same level as the mesozooplancton fraction values, while the mean δ^15^N of BFT larvae from the W-GOM are within the microzooplankton level. Maximum mean values of δ^13^C were found in the lower levels of the size-fractionated groups in the MED, whereas the lowest δ^13^C values belonged to the MED mesozooplankton biomass fraction (Fig 8).

## Discussion

### BFT Spawning scenarios

Though much progress has been made in the past several years in the knowledge of BFT tuna populations, studies have mainly been focused on defining stock identity and structure through genetics, tagging studies[56], [57] and stable isotope markers in otoliths (δ^13^C, δ^18^O) [58]. However, in regard to the early life history of BFT, knowledge has only increased on BFT spawning habitat preferences and on climatic effects on larval growth and condition [59], [10], [11], [60], [13].

The GOM and the MED spawning regions have kinetic energy being supplied by mesoscale structures that cause eddy and frontal formations [28], [9], [10], [60], [14]. Both regions are also characterized by markedly oligotrophic waters, although surface chlorophyll and overall productivity is higher in the offshore GOM compared to the MED [28], [12], [60]. This strategy of spawning in nutrient-poor waters could be developed to overcome predation pressure on larvae[61], [62], [14]. Secondary productivity also appeared to be higher in the GOM than in the MED, with higher mesozooplankton biomass. However, no significant differences were observed in the microzooplankton biomass.

Stable isotope values reported in this study for micro- and meso-zooplankton are in the same ranges as those previously reported for both, the GOM and MED regions [36], [63], [64], [65], [39], [66]. However, despite having similar microzooplankton biomasses, δ^15^N microzooplankton signatures from the W-GOM were significantly lower than in the E-GOM and MED regions. These rather depleted δ^15^N values could be the outcome of diazotrophic N_2_ fixation from atmospheric sources. Cyanobacteria such as *Trichodesmium* spp fix atmospheric nitrogen (N_2_) at high rates and thereby contribute to new production in nutrient depleted waters [67]. *Trichodesmium* is abundant in oceanic stratified warm waters of subtropical regions [68], [69]. This genus is commonly observed, although with highly variable abundances, in the Gulf of Mexico [70], [71], [72].

Nitrogen limitation and the remineralisation of organic matter in microbial food webs may be responsible for the lower δ^15^N enrichment of the plankton size-classes. Rapid consumption of substrates would prevent the discrimination of light versus heavy nitrogen isotopes and the composition of prey and predator would converge [44]. [73] Conclude that N^2^ fixation by *Trichodesmium* is quantitatively significant in the eastern GOM, whereas in the western GOM over Campeche Canyon, high *Trichodesmium spp*. occurrence appears related to geostrophic front associated to cyclonic and anticyclonic circulation observed below the thermocline [74].

The lowest values for microzooplankton δ^13^C were observed in the E-GOM region. The δ^13^C signatures of consumers are determined by the δ^13^C values of primary producers. Elevated δ^13^C values might reflect a carbon source derived from benthic primary producers in addition to phytoplankton [75], thus a carbon with more coastal or inshore origin source [76], [77], [39].

### BFT larval morphometrics

BFT rearing experiment showed that maternal stable isotope signatures are transmitted to the eggs, and progressively to the early larvae of BFT [52]. This maternal signature was discernible until larvae attained postflexion stages. Therefore, this study only considered postflexion stages of BFT larvae, when these are fully capable of preying on a wide range of planktonic organisms, and hence avoiding the effect of maternally transmitted stable isotope signatures.

BFT larvae from the GOM showed significantly lower DW in relation to SL than the MED. This implies region-specific differences in larval growth or condition patterns. Surface temperatures in the GOM were higher than in the MED, suggesting that in the presence of sufficient food BFT larvae in the GOM may grow faster [59].

Moreover, since the total amount of C and N are a consequence of protein synthesis and lipid/fatty acids, C:N ratios can be used to assess the nutritional status [78], [79], [80], [81]and as a proxy for lipid content [82], [83], [84]. The significantly higher C:N ratios of the GOM BFT larvae with respect to the MED larvae seem to indicate differentiated larval growth strategies which may represent the species’ adaptation to the environmental conditions of each spawning area.

### BFT larval trophodynamics

In this comparative study, MED BFT larvae had significantly greater δ^15^N values in comparison to the GOM larvae. Within the GOM, E-GOM larvae had higher δ^15^N values than the W-GOM.This was likely due to differences in the characteristics of water masses among regions, as was shown by a smaller scale SIA study of larval bullet tuna in the Balearic Sea [39].

BFT larvae from the GOM also did not show nitrogen enrichment compared to the micro and mesozooplankton. Rather, δ^15^N signatures for larval BFT from the GOM showed similar trophic level to the zooplankton. This result does not seem to reflect the traditional grazing food chain of phytoplankton-> micro- and mesozooplankton-> fish larvae. However, the average isotopic signatures for δ^15^N of the E-GOM BFT larvae fall within the range of some of the larval and juvenile apex predator species analyzedby [63], such as swordfish, *Xiphias gladius* and the dolphin fishes, *Coryphaena hippurus and C*. *equiselis*, suggesting that this pattern may be characteristic of the GOM region.

The low nitrogen signature of larvae in the GOM could be reflecting the particular prey items and nutrient sources on which larvae are feeding, and where those prey items are in the food chain. It is possible that alternative grazing pathways may be occurring, as a result of the assimilation of N_2_ fixation by the cyanobacterium *Trichodesmium* sp. in the GOM food web. [64] Hypothesized that organisms collected from areas where *Trichodesmium* is abundant will retain significantly depleted δ^15^N values compared to those in areas where concentrations of this cyanobacterium are low, as a result of diazotrophic inputs by *Trichodesmium*.

In this study, isotope signatures were determined for the whole micro- and mesozooplankton populations. However, δ^15^ N signatures of individual zooplankton taxa can range widely. Selective feeding by larvae could therefore result in different δ^15^ N signatures than if larvae were feeding indiscriminately. [34] found that prior to piscivory, *Thunnus* spp. larvae collected in the Straits of Florida had a mixed diet of crustaceans and appendicularians. Consumption of appendicularians by *Thunnus* spp. increased with larval length to a maximum when nearly 80% of the larvae 7–11 mm body length had appendicularians in their guts, suggesting a preferential behavior for consuming this gelatinous zooplankton despite the availability of several types of prey. *Thunnus* spp. may rely on appendicularians as a nutritional “place holder” in an oligotrophic habitat as they try to manage energy demands of fast growth and warm temperatures [34]. Appendicularians are rather ubiquitous in warm oligotrophic regions, and often numerically dominate mesozooplankton assemblages [85]. They filter and ingest small particles using a mucous, external filtering device with small pores, which favors feeding on nanoand picoplankton [86]. Since appendicularians in the W Mediterranean are consumed by BFT larvae [32], and the δ15 N levels of this zooplankters are relatively high in this region with respect to other zooplankton groups (cladocera, copepods or microplankton) [65], the higher δ^15^ N signature in larval BFT in the MED may therefore result from a diet rich in appendicularians.

Lastly, another explanation for the lower GOM nitrogen isotopic values could be the decrease of δ^15^N with temperature. [87] found in cladocerans (*Daphnia magna*) and amphipods (*Hyalella* spp.) consuming identical diets that their body-averaged δ^15^N values declined dramatically with increasing temperatures. The potential causes for the decline included decreasing nitrogen excretion with increasing growth and reduced nitrogen assimilation efficiencies with increased metabolism. A nitrogen trophic fractionation decrease with increased temperature in *Dicentrarchus labrax*has been reported [88], explaining that temperature accelerates most physiological processes [89]and thus higher growth rates are achieved. Higher growth rates lead to reduced excretion and, therefore, increased retention of the lighter isotope is expected. This would result in an inverse relationship between δ^15^N and growth rate.

### Ontogenic feeding shifts

Protistan consumers were not enriched in δ^15^N relative to their prey [90], demonstrating an isotopic invisibility of protozoan trophic steps in marine food webs. This fact can help to explain the δ^15^N ontogenic dynamics observed in this study, with no relationship between BFT larvae δ^15^N values with SL in the three ecosystems studied (E-GOM, W-GOM and MED). While direct observation on stomach content analysis revealed piscivory of most scombrid larvae studied ([34], [31], the expected increase in δ^15^N assumed by piscivory is not shown by our results, probably because of the absence of isotopic enrichment in the microbial food web whose role is important in BFT larval trophodynamics. The observations of this study are consistent with previously reported results for BFT larvae [32], [91], [92], although contrary to hypotheses that BFT larvae overcome the constraints of developing in particularly unproductive waters by relying on cannibalism [29], [93].

The δ^13^C signatures of BFT larvae were distributed over a rather narrow range of values. Differences were mainly observed in the smaller larvae from the MED region, which showed significantly lower δ^13^C values. While MED larvae showed an increasing trend with size of δ^13^C signatures, the GOM larvae showed a decrease with size, suggesting changes in the carbon sources from neritic to oceanic and vice-versa[37], [62].

### Comparative trophic position

Characterizing the δ^15^N values at the base of marine food webs is troublesome because primary producers have short life spans that respond quickly to fluctuations in biogeochemical and physical forces, and concurrently can be difficult to isolate from other organic suspended particulate material. An alternative approach is to use a primary consumer (e.g. zooplankton) as the isotopic reference, i.e. a proxy for the base of the food web, representing the trophic position 2 or slightly higher (e.g. [42]). Primary consumers integrate the phytoplankton isotopic signal over a longer term, reducing the uncertainty in trophic position estimation of consumers located higher in the food web [94], [95], [42]. The use of the microzooplankton baseline for estimating the TP of each BFT larval group seemed reasonable for these regions comparisons because this zooplankton size fraction comprises most of the organisms described in larval tuna diets, such as nauplius stages, copepodites, cladocerans and appendicularians [96], [32], [33], [34]. The highest trophic enrichment and therefore TP among the three regions corresponded to the MED BFT, implying a greater trophic specialization and a greater trophic niche of these larvae ([97], [98] that can result in a major nitrogen efficiency through the food webs [99]. This hypothesis is in accordance with the observation that more productive ecosystems show lower relative trophic levels [100], [101]. Note that the primary consumers base line (microzooplankton) are identical for the MED and E-GOM and significantly higher than the W-GOM (Fig 8).

## Conclusions

This comparative trophic study is based on the N and C stable isotopes of BFT larvae from the main GOM and MED BFT spawning grounds. The environmental scenarios in which these larvae develop showed significant differences in temperature, and in nutrient inputs. The GOM spawning area was warmer (around 3–4°C) in comparison to the MED waters. Such temperature differences can have important consequences for the metabolic rates of BFT larvae, and consequently on the larval growth patterns. Although both regions are typically oligotrophic, the GOM waters, in general, were more productive, particularly in terms of mesozooplankton biomass.

With regards to the stable isotope signatures, the main difference observed was the low δ^15^N of the micro- and mesozooplankton in the W-GOM, which resulted inlowδ^15^N values of BFT larvae in this region. This low trophic positioning of the GOM BFT larvae suggests that they are not part of the traditional food chain from micro- and mesozooplankton to larvae. Unlike the GOM, the MED BFT larvae showed the highest δ^15^N and TP values, well above the underlying microzooplankton baseline. The depleted δ^15^N microzooplankton and larval signatures of the W-GOM may be explained by the rather abundant protists responsible for the fixation of atmospheric nitrogen, bringing new N in the lower levels of the food web and thereby, decreasing the amount of δ^15^N. BFT larvae from the GOM and MED showed opposed ontogenetic diet shifts with growth, with a significant linear increase inδ^13^C in MED larvae with SL, and a linear decrease in GOM larvae.

These results suggest that the food webs and consequent larval ecology among the two spawning sites is distinct. This has implications for the effects of environmental variability and change on larval survival and recruitment among the two stocks, and on the contribution of each stock to overall population recruitment. Continuing research is crucial for understanding larval growth strategies and condition between the two spawning sites, their competition for feeding resources and their exposure to larvae of co-occurring apex predator species, all of which could influence larval survival and recruitment.

## Supporting Information

S1 Table Geographic coordinates of the sampling stations from GOM and MED. (DOCX)
