# Correction: Trophic Ecology of Atlantic Bluefin Tuna (*Thunnusthynnus*) Larvae from the Gulf of Mexico and NW Mediterranean Spawning Grounds: A Comparative Stable Isotope Study

**DOI:** 10.1371/journal.pone.0138638

**Published:** 2015-09-16

**Authors:** Raúl Laiz-Carrión, Trika Gerard, Amaya Uriarte, Estrella Malca, José María Quintanilla, Barbara A. Muhling, Francisco Alemany, Sarah L. Privoznik, Akihiro Shiroza, John T. Lamkin, Alberto García

The species *Thunnus thynnus* is misspelled in the article title. The correct title is: Trophic Ecology of Atlantic Bluefin Tuna (*Thunnus thynnus*) Larvae from the Gulf of Mexico and NW Mediterranean Spawning Grounds: A Comparative Stable Isotope Study. The correct citation is: Laiz-Carrión R, Gerard T, Uriarte A, Malca E, Quintanilla JM, Muhling BA, et al. (2015) Trophic Ecology of Atlantic Bluefin Tuna (*Thunnus thynnus*) Larvae from the Gulf of Mexico and NW Mediterranean Spawning Grounds: A Comparative Stable Isotope Study. PLoS ONE 10(7): e0133406. doi:10.1371/journal.pone.0133406
