# Supplementary material for: Trophic Ecology of Atlantic Bluefin Tuna (Thunnusthynnus) Larvae from the Gulf of Mexico and NW Mediterranean Spawning Grounds: A Comparative Stable Isotope Study
Source: PLoS One. 2015 Jul 30;10(7):e0133406. doi: 10.1371/journal.pone.0133406 (PMC4520599; doi:10.1371/journal.pone.0133406)
Supplement: S1 Table — (DOCX) [file pone.0133406.s001.docx]

**S1 Table. Geographic coordinates of the sampling stations from GOM and MED.**

| **Gulf of Mexico (GOM)** | | | | | | **NW Mediterranean (MED)** | | | | | |
| --- | --- | --- | --- | --- | --- | --- | --- | --- | --- | --- | --- |
| **N** | **Longitude** | **Latitude** | **N** | **Longitude** | **Latitude** | **N** | **Latitude** | **Longitude** | **N** | **Latitude** | **Longitude** |
| 1 | -84,500 | 25,008 | 65 | -91,007 | 27,499 | 1 | 39,3327 | 2,5250 | 63 | 38,1632 | 2,2962 |
| 2 | -85,014 | 24,993 | 66 | -91,001 | 26,992 | 2 | 39,3145 | 2,3018 | 64 | 38,1687 | 2,5195 |
| 3 | -85,506 | 24,986 | 67 | -90,987 | 26,504 | 3 | 39,4888 | 2,2925 | 65 | 38,3339 | 2,7355 |
| 4 | -85,498 | 24,671 | 68 | -90,990 | 25,997 | 4 | 39,6617 | 2,3005 | 66 | 38,3320 | 2,5163 |
| 5 | -84,995 | 24,494 | 69 | -91,496 | 26,001 | 5 | 39,6697 | 2,0845 | 67 | 38,4965 | 2,5165 |
| 6 | -84,486 | 24,502 | 70 | -91,986 | 25,999 | 6 | 39,5000 | 2,0858 | 68 | 38,6696 | 2,5210 |
| 7 | -83,984 | 24,001 | 71 | -92,002 | 26,502 | 7 | 39,3317 | 2,0858 | 69 | 38,8360 | 2,5225 |
| 8 | -83,486 | 23,991 | 72 | -92,002 | 27,006 | 8 | 39,1708 | 2,0787 | 70 | 39,0022 | 2,5187 |
| 9 | -83,491 | 24,498 | 73 | -92,501 | 27,002 | 9 | 39,1670 | 1,8582 | 71 | 38,9997 | 2,7385 |
| 10 | -83,998 | 24,505 | 74 | -92,987 | 27,007 | 10 | 39,3332 | 1,8588 | 72 | 38,8320 | 2,7394 |
| 11 | -83,992 | 24,994 | 75 | -92,998 | 26,495 | 11 | 39,4927 | 1,8663 | 73 | 38,6655 | 2,7339 |
| 12 | -83,999 | 25,504 | 76 | -92,988 | 26,286 | 12 | 39,4933 | 1,6572 | 74 | 38,5026 | 2,7352 |
| 13 | -84,005 | 25,992 | 77 | -93,510 | 26,020 | 13 | 39,3346 | 1,6498 | 75 | 38,4986 | 2,9542 |
| 14 | -84,500 | 26,003 | 78 | -93,992 | 26,018 | 14 | 39,1610 | 1,6571 | 76 | 38,6620 | 2,9555 |
| 15 | -84,997 | 25,995 | 79 | -93,997 | 26,497 | 15 | 39,1690 | 1,4322 | 77 | 38,8361 | 2,9545 |
| 16 | -84,994 | 26,498 | 80 | -93,985 | 27,001 | 16 | 39,3357 | 1,4322 | 78 | 39,0083 | 2,9563 |
| 17 | -84,997 | 27,003 | 81 | -94,497 | 26,999 | 17 | 39,3283 | 1,2243 | 79 | 39,0025 | 3,1733 |
| 18 | -84,996 | 27,497 | 82 | -94,499 | 27,081 | 18 | 39,3333 | 0,9990 | 80 | 38,8321 | 3,1681 |
| 19 | -84,997 | 27,996 | 83 | -94,413 | 26,999 | 19 | 39,1675 | 0,7761 | 81 | 38,6687 | 3,1678 |
| 20 | -85,495 | 28,677 | 84 | -94,506 | 26,907 | 20 | 39,0072 | 0,7766 | 82 | 38,4988 | 3,1772 |
| 21 | -86,002 | 29,187 | 85 | -94,608 | 27,002 | 21 | 38,8332 | 0,8085 | 83 | 38,3424 | 3,1770 |
| 22 | -86,494 | 29,500 | 86 | -95,006 | 26,989 | 22 | 38,6663 | 0,7852 | 84 | 38,4961 | 3,3904 |
| 23 | -86,500 | 28,992 | 87 | -95,007 | 26,497 | 23 | 38,5041 | 0,7800 | 85 | 38,6673 | 3,3879 |
| 24 | -86,002 | 28,505 | 88 | -95,012 | 26,021 | 24 | 38,3367 | 0,7805 | 86 | 38,8353 | 3,3913 |
| 25 | -85,995 | 27,994 | 89 | -95,490 | 26,018 | 25 | 38,1703 | 0,7836 | 87 | 39,0023 | 3,3965 |
| 26 | -86,005 | 27,506 | 90 | -95,998 | 26,027 | 26 | 38,0024 | 0,7850 | 88 | 39,1658 | 3,3960 |
| 27 | -85,996 | 26,994 | 91 | -96,008 | 26,501 | 27 | 38,0024 | 0,9989 | 89 | 39,1652 | 3,1753 |
| 28 | -85,997 | 26,495 | 92 | -95,999 | 26,997 | 28 | 38,1637 | 0,9991 | 90 | 39,1648 | 3,6082 |
| 29 | -86,014 | 26,002 | 93 | -95,996 | 27,550 | 29 | 38,3305 | 1,0010 | 91 | 38,9979 | 3,6086 |
| 30 | -85,994 | 25,490 | 94 | -95,992 | 27,994 | 30 | 38,4987 | 0,9958 | 92 | 38,8357 | 3,6083 |
| 31 | -86,003 | 24,985 | 95 | -95,495 | 27,999 | 31 | 38,6721 | 1,2134 | 93 | 38,6692 | 3,6065 |
| 32 | -86,450 | 25,498 | 96 | -95,011 | 28,006 | 32 | 38,4914 | 1,2154 | 94 | 38,8360 | 3,8393 |
| 33 | -87,491 | 26,007 | 97 | -94,495 | 28,009 | 33 | 38,8360 | 1,0002 | 95 | 39,0013 | 3,8284 |
| 34 | -87,998 | 26,000 | 98 | -93,487 | 27,997 | 34 | 38,3592 | 1,2101 | 96 | 39,1660 | 3,8258 |
| 35 | -88,996 | 26,015 | 99 | -91,996 | 28,003 | 35 | 38,1663 | 1,2034 | 97 | 39,5012 | 3,3858 |
| 36 | -89,495 | 25,992 | 100 | -90,999 | 27,997 | 36 | 38,0057 | 1,2180 | 98 | 39,3345 | 3,3771 |
| 37 | -89,008 | 26,504 | 101 | -89,845 | 27,672 | 37 | 38,1707 | 1,4301 | 99 | 39,3332 | 3,6039 |
| 38 | -88,999 | 26,995 | 102 | -89,513 | 27,510 | 38 | 38,3351 | 1,4321 | 100 | 39,3315 | 3,8187 |
| 39 | -88,498 | 27,001 | 103 | -88,993 | 27,155 | 39 | 38,4988 | 1,4279 | 101 | 39,4978 | 3,6047 |
| 40 | -87,998 | 27,001 | 104 | -88,758 | 27,171 | 40 | 38,6691 | 1,6515 | 102 | 39,4917 | 3,8283 |
| 41 | -88,001 | 26,502 | 105 | -88,504 | 27,166 | 41 | 38,8306 | 1,6487 | 103 | 39,6657 | 3,8222 |
| 42 | -87,001 | 26,287 | 106 | -88,254 | 27,170 | 42 | 38,8345 | 1,8683 | 104 | 39,8338 | 3,8280 |
| 43 | -86,996 | 26,507 | 107 | -88,492 | 26,999 | 43 | 38,6689 | 1,8516 | 105 | 39,8297 | 4,0415 |
| 44 | -87,000 | 27,000 | 108 | -88,490 | 27,081 | 44 | 38,5007 | 1,8690 | 106 | 39,6675 | 4,0370 |
| 45 | -86,996 | 27,497 | 109 | -88,589 | 27,161 | 45 | 38,5049 | 1,6512 | 107 | 39,5117 | 4,0427 |
| 46 | -87,000 | 28,015 | 110 | -88,504 | 27,250 | 46 | 38,3345 | 1,6578 | 108 | 39,3317 | 4,0363 |
| 47 | -87,011 | 28,497 | 111 | -88,408 | 27,171 | 47 | 38,3338 | 1,8740 | 109 | 39,1742 | 4,0332 |
| 48 | -86,992 | 29,008 | 112 | -88,432 | 27,414 | 48 | 38,1650 | 1,8730 | 110 | 39,0042 | 4,0378 |
| 49 | -86,998 | 29,981 | 113 | -88,599 | 27,578 | 49 | 38,3462 | 2,0987 | 111 | 39,1767 | 4,2578 |
| 50 | -88,873 | 27,500 | 114 | -88,662 | 27,750 | 50 | 38,5023 | 2,0833 | 112 | 40,1733 | 4,5814 |
| 51 | -88,702 | 27,496 | 115 | -88,752 | 27,908 | 51 | 38,6712 | 2,0838 | 113 | 39,6677 | 4,2669 |
| 52 | -88,513 | 27,501 | 116 | -88,250 | 27,994 | 52 | 38,8367 | 2,0863 | 114 | 39,5018 | 4,2541 |
| 53 | -88,318 | 27,502 | 117 | -88,010 | 27,991 | 53 | 39,0048 | 2,0892 | 115 | 39,5010 | 4,4750 |
| 54 | -88,129 | 27,503 | 118 | -87,656 | 27,996 | 54 | 39,3333 | 2,7365 | 116 | 39,3358 | 4,2567 |
| 55 | -88,256 | 27,006 | 119 | -87,420 | 28,010 | 55 | 39,1657 | 2,7387 | 117 | 39,3318 | 4,4783 |
| 56 | -88,499 | 26,999 | 120 | -87,169 | 28,003 | 56 | 39,1702 | 2,5245 | 118 | 39,1705 | 4,4753 |
| 57 | -88,743 | 26,996 | 121 | -87,005 | 28,171 | 57 | 39,1680 | 2,3159 | 119 | 39,0037 | 4,2594 |
| 58 | -89,995 | 25,999 | 122 | -86,750 | 28,256 | 58 | 38,9995 | 2,3021 | 120 | 38,8205 | 4,0412 |
| 59 | -90,000 | 26,499 | 123 | -86,590 | 28,428 | 59 | 38,8337 | 2,3040 | 121 | 38,6671 | 3,8257 |
| 60 | -89,990 | 27,000 | 124 | -86,828 | 28,740 | 60 | 38,6661 | 2,3027 | 122 | 38,5018 | 3,6103 |
| 61 | -90,490 | 27,008 | 125 | -86,995 | 28,753 | 61 | 38,5067 | 2,2993 | 123 | 38,3433 | 3,3867 |
| 62 | -90,505 | 27,243 | 126 | -87,248 | 28,666 | 62 | 38,3358 | 2,3056 | 124 | 38,1687 | 3,1688 |
| 63 | -90,697 | 27,241 | 127 | -87,577 | 28,912 |  |  |  |  |  |  |
| 64 | -90,693 | 27,498 |  |  |  |  |  |  |  |  |  |
